# An Essay on the Nature and Cure of Insanity, with Observations on the Rules for the Detection of Pretenders to Madness

**Published:** 1816-02

**Authors:** 


### PART II.

### COMPREHENSIVE ANALYTICAL REVIEW OF MEDICAL LITERATURE.

"Tros, tyriusve, *nobis* nullo discrimine agetur."

*An Essay on the Nature and Cure of Insanity, with Observations on the Rules for the Detection of Pretenders to Madness.* By GEORGE NESSE HILL, Medical Surgeon, &c. 1 vol. 8vo. pp. 446. London, 1814.

THIS publication should have been noticed earlier; but, as it is the intention of the Medico-Chirurgical Reviewers not to let any work of importance pass without delineating its contents to the public, they take this opportunity of ushering Mr. Hill's performance into the medical world, though it is with some trepidation, for Mr. Hill is a very learned gentleman, and a sturdy champion of the opinions he has embraced. The object of the work before us appears, from the author's own words, to be no less than that of acquiring the gratifying title of "friend to the human race;" next to which, is the ambition of leaving some memorial of his name,— not steal inglorious to the silent grave." xi. Whether these objects shall be gained by the volume under consideration, remains to be proved; but we would just hint, that those works which have stood the test of time, and come down with undiminished reputation from age to age, do not owe their *immortality* to the adroitness with which their authors linked together a chain of *quotations from others,* constituting three-fourths of their contents. Here Mr. Hill has erred lamentably; for all that niggardly futurity will ever bestow on such writers is the merit, or rather the labour, of "great reading."

Mr. Hill complains, perhaps with justice, of the unsatisfactory information which the volumes that have hitherto been written on insanity convey to the enquiring mind. We sincerely wish that the work before us will, as the author hopes, prevent the repetition of such expressions of regret in future; but we fear that after what has been written and done with so little success by so many practical authors, in respect to insanity, it will require a greater genius than Mr. Hill to persuade the faculty —" that insanity is as generally curable as any of those violent diseases *most successfully* treated by medicine." xii.

The scope of our author's Essay may be learnt from the following deductions: — 1st. That insanity has always corporeal disease for its foundation. 2d. That it consists of but one species under two forms, viz. the STHENIC and the ASTHENIC, or Mania and Melancholia. 3d. That it is not an hereditary disease in the vulgar sense of the word, as commonly understood. 4th. That it is as generally curable, &c. vide sentence quoted above. We shall follow the author in his own divisions.

The first chapter, of fifty-three pages, contains at least thirty pages of quotations, from every work that comes in our author's way, from Hippocrates to Haslam; from Locke on the Understanding to *Godwin on Political Justice!* to prove that mind is matter, and matter mind:— " all *brainular* affections, whether denominated insane or otherwise, tend most decidedly to prove that mind and body are *homogeneous*." 35. It is true that Mr. Hill will not quarrel with us about a *soul;* we are at liberty to have as many of these as we please; but if we attempt to maintain the immateriality of mind, we shall immediately be branded with ignorance and hypocrisy.

" Doubtless, those men [meaning himself as one of course] who are the firm and faithful votaries of truth will, in all ages, be attacked by ignorance and persecuted by hypocrisy; but the triumph of their cause is for ever secured." 53.

We shall not, therefore, enter the lists with our author upon this occasion, but leave him to that " cold and comfortless system of materialism," of which he is evidently the champion, for it is an insult to our understandings to first level intellect in the dust, and then tell us we need not be alarmed about a soul, since *scripture* has assured us on that point! And to what end, after all, has this elaborate disquisition of Mr. Hill's tended? We all knew, before his work appeared, that the *manifestation* of mind depended entirely on organization, for who ever supposed that a man could think without brains? In this way, we can very easily suppose that mental derangement results from corporeal derangement, without identifying mind and matter. When the corporeal fabric, and particularly the brain, deviates from a state of sanity, the mind can no more *manifest* itself *sanely*, than a musician can bring harmonious tones from an untuned harpsichord. Now this theory is fully as applicable to every practical purpose in the investigation or treatment of insanity as the system of mental materialism of our author; and why, therefore, should we wantonly drag away any prop from the fabric of religion, when the displacement tends to no use, and the edifice itself is of the utmost importance, even in a temporal view, to the happiness and prosperity of mankind.

"CHAP. II. Insanity consists of one species under two forms, viz. Sthenic and Asthenic, or Mania and Melancholia."—

We must confess that the following sentence, which opens this chapter, did not prepossess us much in favour of Mr. Hill's judgment, notwithstanding the "long experience" which he has had.

"Two families of disease comprehend the whole, says a *young* ingenious writer, in the Medical and Physical Journal; and he has said rightly, whatever reluctance may exist in the minds of *some medical practitioners* to allow the justness, truth and precision of this simple but solid fact." 54.

If Mr. Hill's "longings after immortality" have no surer grounds of hope than this brilliant hypothesis of the young Medical and Physical writer, we much fear that the *paper* of his book, and the *intellectual* effusions imprinted thereon, are doomed, like *mind and body*, in his favourite system, to a certain "*homogeneous*" decay; and consequently to oblivion! But we will not augur so badly; for we hope, that after wading through the deserts of conjecture, we shall occasionally meet an oasis of facts or useful reasonings, that may remunerate our research, and preserve the volume from neglect.

"Insanity affords no exception to this arrangement; (sthenic and asthenic) every lunatic belongs to the high or low form. By the sthenic and asthenic, nothing more is meant than the opposite states of excitability of the system at large at the period of insane attack." 56.

The medical atmosphere of our author's ideas is so constantly darkened by the clouds of quotations, which sail on the four winds from every quarter of the literary compass, that it is with much difficulty we can comprehend his own personal opinions, and especially in this chapter, respecting the excitablity of the system in maniacs. If we are not mistaken, he conceives, that excitability is torpid or deficient in melancholia, and too vivid or superabundant in mania; he also hints, that in certain cases, there is an unequal distribution of the excitability; all which observations may be applied to numerous other diseases as well as to insanity; and in fact, do not bring us one whit nearer to a knowledge of the real state of the corporeal organs in the latter disease, than we were before Mr. Hill published his elucidation. For example:—

"Certain *peculiar* states of the general excitability, and of the brain in particular, are inevitably followed by a *correspondent* mental deranged state; nor can this state become altered to another, however slight the difference, without a similar mental change in perfect unison with it. Now, that the brain is liable to enter into a state favourable to the presence of insanity from impressions made upon it through the medium of diseased visceral organs, is most certainly known to unerring experience; consequently, the event will follow, by inducing a correspondent state of mind; but with regard to the *exact physical nature of this change or peculiar state, and how effected, seems to be placed far beyond mortal ken.*" 58.

Thus, after all the fuss about the materiality of the mind, and the corporeal nature of insanity, we are brought back to the point from whence we set out—that we know little or nothing about the matter! Perhaps, indeed, the following luminous passage may unravel the mystery.

"Sthenic diathesis, when connected with insanity, corresponds with a state of *too great mobility of brainular action*, or the strong nervous excitement of M. Pinel, and of the whole nervous system; hence, while under this state of action, persons are *not* exposed to the consequences, though they *may be to the influence* of the depressing passions, they are not only proof *against such influence*, but are carried incessantly onward in despite of their most vigorous attack." 60.

Whether it may be owing to the gross *materiality* of *our* minds, we cannot say, but certainly we have more than twice or thrice read over this, and various other passages in the volume before us, without being able to decypher our author's meaning. In the 63d page, Mr. H. flies into a paroxysm of rage—we had almost said, insanity, because Mr. Haslam has dared to write a passage which is rather in opposition to his favourite sthenic and asthenic creed.

"I would strongly oppose," says Mr. Haslam, "their (mania and melancholia) being considered as opposite diseases, they appear to differ only from the different passions which accompany them; on dissection, the state of the brain does not shew any appearances peculiar to melancholy, nor is the treatment which I have observed most successful, different from that which is employed in mania."

On this passage of Mr. Haslam, simply setting forth the result of experience and ocular observation, our champion for truth and materialism coolly asserts, "that it is a most *unfounded* and pernicious statement."

Now we appeal to the faculty, whether Mr. Hill does not exhibit the very height of illiberality in thus using so coarse an expression as almost "the lie direct," to a practical gentleman, who without any theory to support, is merely stating the result of his observations! These, however, are the champions for *truth*, who knock down, or charge with falsehood, every one who ventures to dissent from them!

### History of the Sthenic, or high Form of Insanity.

From passages like the above, we gladly turn to those which are more honorable to our author, and more useful to the public. The present Section exhibits an animated and well drawn portrait of Sthenic Insanity, from which we shall extract the more prominent traits, principally in the energetic words of Mr. Hill.

The eye frequently denotes slow approaching insanity of the sthenic form, before any other feature. Patients themselves have described a sensation as if fire flashed from the organ. A great majority of the author's patients had the oculus bovinus, or protruding eye. Perpetual motion or roving in every direction marks the first attack. During the paroxysm, the ball of the eye is stiffly and firmly pushed forward; the pupils contracted; the adnata bright and tense; sometimes yellow, and streaked with turgid blood-vessels. During extreme excitement, the vessels are so distended as to elevate the palpebræ. The muscles of the face acquire a new character of action, while the fleeting passions and quick succession of ideas, are painted with an evanescent rapidity that defies the pen or the pencil. The whole muscular powers of the body early feel the influence of insanity, acting with preternatural vigour and agitation. Even in sleep the sthenic patient's muscle retains a rigidity manifesting unremitting vigilance. Generally speaking, this description of lunatics are insusceptible to the extremes of heat and cold, and also to the influence of contagious diseases—even hydrophobia is suspended during the insane paroxysm. Torpor of the skin, stomach and bowels prevails. The attack of sthenic insanity is sometimes instantaneous, at least its manifestation. The brain, although most commonly the seat of derangement, is not exclusively so, every important viscus appearing occasionally to be the source of the mischief.

"Sometimes," says Struve, "the vital principle is immoderately active in an individual organ, by which the whole system suffers, and the necessary life is withdrawn from the other organs."

Hence diminished action of the stomach and bowels; unusual fulness; pain of distension; stricture, or corded feel of the whole head; vertigo; flushing of the face alternating with plumbean pallor, mark a preternatural determination to the head; all which appearances are subject to much quicker changes than in true *phrenitis.* Indistinct vision; sudden loss of memory; pervigilium sometimes indicate the attack.

After describing the terrific symptoms of the maniacal paroxysm itself, Mr. Hill observes, that wherever *pain* resides, it is at times excruciating; and even when the consciousness of it is absorbed, the muscular action excited for its removal is very great, and highly characteristic of the true nature of the disease. Sometimes the seat of the pain will be pointed out by the patient himself. When entirely unrestrained, and the pain great, the maniac appears to have an irresistible impulse to run off in any direction of the compass to which his eyes happen to be directed. At this awful moment, woe be to the unfortunate relation that has incurred his resentment; for if armed with any missile weapon, the life of the supposed enemy is in imminent danger. Maniacs of this description rarely commit suicide, Melancholics often.

The cutaneous glands and materia perspirabilis become altered, the latter disagreeably so. The eructations from the stomach have a peculiar fœtor, resembling that evolved from the leaves of the hyoscyamus niger. Inequality of temperature is marked by a burning heat, with cold, often clammy feet, and *vice versa.* The pulse is so variable, that nothing can be depended on from observing it.

"In almost all cases of insanity, an increase of the pulse is observable at times, not indeed enough to account for delirium, still enough to prevent its being said with propriety, that it is without fever."

The urine is high coloured, the saliva scanty and creamy. Tongue has usually a yellowish white list running down the centre. Appetite capricious, often voracious. In females, the pain (always present somewhere) is often seated in the uterus, in the præcordia, or hepatic or splenic regions. In males, the hypochondres or thorax are often the seat of pain; but in both sexes, the head is the more usual part affected. And though the most excruciating pain may exist in a part for years, dissection rarely detects any organic alteration of structure. Sudden emaciation is rather the effect of art than disease. Delirium is the most terrific symptom of all, and very generally attendant on this form of insanity.

*Termination of Sthenic Insanity.*

These are, according to our author, melancholia; fatuity; paralytic affection; in a few cases, sudden death as from a stroke of apoplexy. The remainder are cured. Mr. Hill asserts, that sthenic is readily converted into asthenic insanity, or even fatuity, by violent evacuations suddenly reducing the strength; long continued detrusion of reason will suspend for years the most obstinate diseases—even phthisis is incompatible with mania. When sudden death occurs, it always succeeds a violent paroxysm of the disease, and appears to be a complete exhaustion of the excitability. Phthisis suspended by insanity in delicate females, returns when the latter disappears. Health is the result of sthenic insanity, when the treatment is early, judiciously, and perseveringly applied. In absolute incurable chronic insanity, although the system seems to have customary healthy functions, yet there is no doubt that visceral disease existed at the beginning, though Nature has now adapted every thing to the change. Original sthenic insanity is ever accompanied with fever, but greatly disposed to become chronic by neglect or improper treatment.

*History of Asthenic Insanity, or Melancholia.*

The middle and advanced periods of life are the most common for the accession of this form, when *tædium vitæ* commences its attack. Debility, with the lesion of function in some important organ, is the foundation of this form of mental aberration, although, from the slow obscurity of the attacks, the visceral derangement is not always early discoverable. Its seat, according to the opinion of Pinel, is almost always in the epigastric region, from whence it is propagated as it were by irradiation. Asthenic lunatics sometimes inflict severe pain upon themselves, or by an irresistible impulse commit mischief upon others, from mechanical action calculated to remove the indescribable misery of the morbid sensation. Intelligent recovered asthenic lunatics describe the onset of the disease as commencing with pain, sometimes fixed, as in the sthenic, but more commonly vagrant; yet in the sum total, grievous, unremitting, and intolerable, resembling the sufferings from great inanition, or the sudden abstinence from long accustomed stimulus. In this situation, the hypochondriac should be attended to, otherwise suicide may probably be the consequence. As pain and diseased impressions advance, reverie and delusive ideas become evolved of a more concentrated nature, and the sufferer is continually dwelling on some mournful topic until general torpor renders him no longer conversible. In some cases, this general torpor is less manifest in the dawn of the malady, than horrid languors, fearful apprehensions, tremblings and startings from impenetrable gloom to ceaseless anguish during the day, and sleepless torment by night. Cutaneous temperature and sensibility become gradually changed; impressions from heat are never mentioned, but those from cold occasionally. Sleep is unequal, and disturbed with uncommonly singular dreams:

> ——— " Should sleep his harrass'd limbs compose,
> And steal him one short moment from his woes,
> Then dreams invade———".

or still more wretched incubus. Reverie becomes daily more profound, accompanied with certain peculiar automatic motions. Dilated pupils, contracting slowly; dull, muddy look of the eye, which rolls heavily on surrounding objects, or is fixed in unmeaning stare on vacancy. The situation of the pain is various, and the degree of violence uncertain. It is rarely in the head primarily, but often secondarily, from visceral derangement, particularly of the liver. The hepatic region in males, the uterine in females, are the common situations of asthenic pain. Hepatic and splenic uneasiness often alternate with pain of the stomach; and the yellow tinge of the countenance will, under these circumstances, be very fugacious. Cold clammy skin and feet, with transient purple-coloured flushings of the face, are common to this form. Evolution of fœtid air (having the peculiar insane odour) takes place, with relief to the indescribably uneasy intestinal commotions and feelings. Preternatural pulsations, extending from the rectum to the œsophagus, are common in the early stages of asthenic insanity, and are extremely distressing to the sufferer, suggesting the idea of being possessed by torturing animals.

> " To whatever cause it be owing (whether to constitution, or God's express appointment), I am hunted by spiritual hounds in the night-season."—*Hayley's Life of Cowper.*

Torpor of the chyliferous system is manifest, while an abundant secretion of cold, ponderous, viscid matter, anciently denominated phlegm, coats the stomach and intestines, rendering them insensible to the action of the strongest medicines. Constipation and flatulence are very troublesome; and diarrhœa rarely appears, unless when the disease is about to undergo a salutary change.

"When the brain is the primary seat of organic lesion, the urine is almost invariably of a whitish limey appearance."

Notwithstanding the general torpor of the system, the asthenic lunatic is considerably under the influence of atmospheric changes. The relaxed scalp is also a symptom of melancholia, and indicative of debility.

### *Terminations of Asthenic Insanity.*

Apoplexy, palsy, epilepsy, dropsy, phthisis, fatuity, chronic lunacy, and suicidal death, are the usual; health, unassisted by art, and natural sudden death, but rarely the terminations. When in apoplexy, *sudden* increase of fluids in the cerebral system appears to extinguish the excitability; but in the majority of cases no vascular distension can be perceived. When in palsy, the insanity generally disappears for a short period previously to the attack. When in epilepsy, the two diseases frequently alternate, epilepsy generally closing the scene. When in phthisis, the subjects are most frequently females, and contrary to the fact in other phthisical cases, they have a decided presentiment that the new disease will terminate fatally.

Fatuity is a termination most frequently met with in males of an originally feeble mental stamina and little sensibility. Chronic insanity is the most frequent termination of all.

Suicide; the detrusion of human reason is so commonly attended by this termination of existence, that Montesquieu observes,

"In England suicide cannot be punished, without punishing the effects of madness."

### *Predisposing and exciting Causes of Insanity.*

"There are no purely *mental* causes of predisposition to insanity," says our author; "all are referable to corporeal derangement. It is the state of the body alone which determines the result of every forcible impression made upon it, and not the converse, or their excitement, which determines the corporeal state."

We do not agree with our author here. When a violent fit of anger or jealousy produces a derangement in the biliary secretion, is it not the mental impression which determines the corporeal state? No, says Mr. Hill—"All human passions are the offspring of impressions made upon the *body*," (114); and consequently, to support my theory, I maintain, that the jealousy or anger was the *result* of the biliary derangement, although prior to it in point of time!

"The concentrated action of the most powerful of the passions, combined with the most horrible circumstances, is incapable of producing insanity, epilepsy, or any other disease, independent of a concurrent corporeal state."

Granted. But then we maintain, that under such circumstances, the mental affections produce the corporeal changes, as under others the corporeal produce the mental. But theory aside, let us return to facts. The skulls of lunatics, though found of every shape, yet in a majority of cases (says our author) they have not a rounded and moderately capacious form, but a narrow contracted one, pinched in at the sides, as if by that abominable article of infantile dress—the forehead-cloth. *Early terror*, operating upon a feeble system, proves a luxuriant source of those ailments termed nervous, and ought to be discountenanced. As youth advances, the predisposition to sthenic insanity is often laid in too high living and habitual stimulation. On the other hand, too great corporeal delicacy often lays the foundation of the asthenic forms.

"Manustupration is a frequent cause of the production of predisposition to madness, appearing first in the shape of early hypochondriasis."

The long protracted imbecile state of the late Danish monarch is said to have arisen from this solitary vice. Impaired digestion and vertiginous symptoms in such subjects, when followed by lapse of memory and confusion of thought, only require a certain concatenation of thought to produce asthenic insanity. Retarded, but especially suddenly suppressed catamenia, have induced predisponent tendency to insanity. Accidental falls or blows on the head will often produce such an early change as to insure future mental aberration.

Too liberal and early a use of diffusible stimulants will predispose to mental derangement.

"The feeble progeny of diseased or enervated parents are often thought to require a glass of what is fancifully called corroborating old Port wine at noon or after dinner. Such indulgence is carried far beyond the medical allowance, until it has become a habit, proving the source of future hypochondriacism, insanity, and suicide." 120.

We quote this passage, because medical men do not set their faces against such practices with sufficient energy, probably from fear of offending the indulgent mamma; but such dereliction is unpardonable. Great inanition, on the other hand, interrupted occasionally by full meals, is equally prejudicial. Severe diseases occurring in early life will often lay the foundation of insanity. Syphilis, and the abuse of its remedy, mercury, will do the same. Intense study is a well-known predisponent cause. A long discussion on the term predisposition to insanity concludes thus:

> " It has long appeared to me, that predisposition to sthenic insanity consists of the presence of that *simple elementary change* termed a state of too great *mobility of the brain and nervous system*, subjecting the whole to be *more readily* excited into positive diseased action by certain causes, than is consistent with the laws of health: the state of predisposition to asthenic insanity being the opposite general situation, or state of immobility." 129.

But surely here is great inconsistency. If *too great* mobility renders the brain and nervous system *more*, surely *immobility* ought to render them *less* " readily excited " into diseased action! Thus the theorist is caught in his own toils: but granting there was no inconsistency, does this *mobility* or *immobility* bring us an inch nearer our object, or convey to us any clearer ideas of the predisposition to, or nature of, insanity? We fear the general answer will be—No.

The section on *proximate cause* of insanity occupies not more than *four* pages, but would require at least as many years to analyse and develope its meaning.

> " Upon considering these facts with all the care they demand, it appears deducible that the *causa proxima* of insanity consists of a *peculiar or specific change in the power of accumulation*, and *subsequent action of the subtle* matter of nervous influence." 132.

We have given this proximate cause of insanity to our readers in the author's own words, lest any part of it should be obscured by our mental — we beg pardon, our *material* faculties. It is true, our " brainular" exertions have been great to comprehend this and many other passages in the work before us; for so *corporealised* are Mr. Hill's *ideas*, when theory is the subject, that they are utterly incapable of transfusion into the *minds* of others. It is therefore with pleasure we read, at the end of this section, that " the subsequent business of the work will consist of considerations less subjected to difference of opinion." Our subsequent labour shall therefore be, the delineation of our author's observations on the exciting causes and treatment of insanity; and although we have had some *dif-*

*ferences* at the commencement of our journey, yet we hope not only to become reconciled as we proceed, but even to part with sentiments of reciprocal friendship and esteem.

(To be concluded in our next.)

